# Evaluation of *IRX* Genes and Conserved Noncoding Elements in a Region on 5p13.3 Linked to Families with Familial Idiopathic Scoliosis and Kyphosis

**DOI:** 10.1534/g3.116.029975

**Published:** 2016-04-12

**Authors:** Cristina M. Justice, Kevin Bishop, Blake Carrington, Jim C. Mullikin, Kandice Swindle, Beth Marosy, Raman Sood, Nancy H. Miller, Alexander F. Wilson

**Affiliations:** * Genometrics Section, Computational and Statistical Genomics Branch, National Human Genome Research Institute, National Institutes of Health, Baltimore, Maryland 21224; † Zebrafish Core, Translational and Functional Genomics Branch, National Human Genome Research Institute, National Institutes of Health, Bethesda, Maryland 20892; ‡ National Institutes of Health Intramural Sequencing Center, National Human Genome Research Institute, National Institutes of Health, Rockville, Maryland 20852; § Department of Orthopedic Surgery, University of Colorado Anschutz Medical Campus, Aurora, Colorado 80045; ** McKusick-Nathans Institute of Genetic Medicine, Johns Hopkins University School of Medicine, Baltimore, Maryland 21224

**Keywords:** idiopathic scoliosis, IRX genes, zebrafish transgenesis, conserved noncoding regions, kyphoscoliosis

## Abstract

Because of genetic heterogeneity present in idiopathic scoliosis, we previously defined clinical subsets (*a priori*) from a sample of families with idiopathic scoliosis to find genes involved with spinal curvature. Previous genome-wide linkage analysis of seven families with at least two individuals with kyphoscoliosis found linkage (*P*-value = 0.002) in a 3.5-Mb region on 5p13.3 containing only three known genes, *IRX1*, *IRX2*, and *IRX4*. In this study, the exons of *IRX1*, *IRX2*, and *IRX4*, the conserved noncoding elements in the region, and the exons of a nonprotein coding RNA, *LOC285577*, were sequenced. No functional sequence variants were identified. An intrafamilial test of association found several associated noncoding single nucleotide variants. The strongest association was with rs12517904 (*P* = 0.00004), located 6.5 kb downstream from *IRX1*. In one family, the genotypes of nine variants differed from the reference allele in all individuals with kyphoscoliosis, and two of three individuals with scoliosis, but did not differ from the reference allele in all other genotyped individuals. One of these variants, rs117273909, was located in a conserved noncoding region that functions as an enhancer in mice. To test whether the variant allele at rs117273909 had an effect on enhancer activity, zebrafish transgenesis was performed with overlapping fragments of 198 and 687 bp containing either the wild type or the variant allele. Our data suggests that this region acts as a regulatory element; however, its size and target gene(s) need to be identified to determine its role in idiopathic scoliosis.

Idiopathic scoliosis is defined as a lateral curvature of the spine greater than ten degrees (°) documented by radiographic analysis, and present in the late juvenile or adolescent period in otherwise normal individuals. The prevalence of idiopathic scoliosis in the general population is estimated to be 2–3% (Bunnell 1986; Lonstein and Carlson 1984). Both sporadic [idiopathic scoliosis (IS)] and familial forms [familial idiopathic scoliosis (FIS)] exist. Several genetic analyses have reported linkage of FIS to various candidate regions, including chromosomes 6p (LOD = 1.42), 10q (LOD = 1.60), and 18q (ATA82B02, LOD = 8.26) (Wise *et al.* 2000); 17p11.2 (D17S799, LOD = 3.2) (Salehi *et al.* 2002); 19p13.3 (D19S922, LOD = 4.087), and 2q (LOD = 1.72) (Chan *et al.* 2002); Xq23-26 (GATA172D05, LOD = 2.23) (Justice *et al.* 2003); 6p25-22 (D6S1031, *P*-value = 0.0032), 6q14-16 (D6S1031, *P*-value *=* 0.0092), 9q32-34 (D9S915, *P*-value = 0.0005), 16q11-q12 (D16S2623, *P*-value = 0.0005) and 17p11-q11 (D16S2623, *P*-value = 0.0005) (Miller *et al.* 2005); 8q12 (D8S1136, LOD = 2.77) (Gao *et al.* 2007); 9q31.2-q34.2 (D9S2157, LOD = 3.64) and 17q25.3-qtel (AAT095, LOD = 4.08) (Ocaka *et al.* 2008); 12p (GATA49D12, LOD = 3.5) (Raggio *et al.* 2009) and 3q12.1 (D3S2462, LOD = 3.01), and 5q13.3 (D5S203, LOD = 3.0) (Edery *et al.* 2011). Population based genome-wide associations of IS have been reported to rs11190870 (*P*-value = 1.24 × 10^−19^) on 10q24.31 near *LBX1* (Takahashi *et al.* 2011), rs6570507 (*P*-value = 1.27 × 10^−14^) on 6q24.1 in *GPR*126 (Kou *et al.* 2013), and rs12946942 (*P*-value = 4 × 10^−8^) on 17q24.3 near *SOX9* and *KCNJ2* (Miyake *et al.* 2013). These findings, some of which have been independently replicated, suggest that IS and/or FIS is a complex genetic disorder with substantial clinical and genetic heterogeneity.

In an effort to reduce the genetic heterogeneity present in FIS, we previously created subsets from our collection of FIS families based on either the most-likely mode of inheritance or on different clinical criteria (Justice *et al.* 2003; Miller *et al.* 2005, 2006). One of our clinically defined subgroups, the kyphoscoliosis (KS) group, consisted of non-Hispanic white families (seven families, 53 individuals) with two or more individuals having a scoliotic curve ≥ 10° in combination with a thoracic curve ≥ 40° (Miller *et al.* 2006). The prevalence of KS in the United States is estimated to be one in 1000 people for lateral and posterior spinal curvatures > 35°, and one in 10,000 for more severe KS, defined by lateral and posterior curvatures > 70° (Bergofsky 1979). Previous linkage analysis of the KS subgroup identified candidate regions on chromosomes 5p15.3, 13q13.3, and 13q32, and analyses of single nucleotide variants (SNVs) narrowed the linkage region on 5p15.3 to about 3.5 Mb (*P*-value = 0.002) (Miller *et al.* 2006). The only genes present in this region are *IRX1*, *IRX2*, *IRX4*, and *C5orf38*, a protein precursor in the promoter region of *IRX2*, which has coordinated expression with *IRX2* and lacks homology to any known protein in the public databases (Wu *et al.* 2006).

The Iroquois (*IRX*) genes are members of a highly conserved gene family, the *TALE* (three-amino acid-loop extension) homeobox genes that encode homeoproteins (Bürglin 1997). Vertebrate *IRX* genes have been found to play a role in neural tube, heart, and ectoderm patterning (Cavodeassi *et al.* 2001; Gómez-Skarmeta and Modolell 2002). In humans, the *IRX* genes are clustered into two groups of three genes believed to be the result of a segmental duplication, because *IRX1*, *IRX2*, and *IRX4* on 5p are paralogs of *IRX3*, *IRX5*, and *IRX6* respectively, on chromosome 16q (Bosse *et al.* 2000; Gómez-Skarmeta and Modolell 2002; Ogura *et al.* 2001; Peters *et al.* 2000). This region on 16q was previously found to be linked to FIS (*P*-value < 0.0005) in a sample of 202 FIS families when the threshold for scoliosis was ≥ 30° (Miller *et al.* 2005).

Our previous linkage results suggest the involvement of the *IRX* gene family and/or transcriptional enhancers within the highly conserved noncoding elements (CNE) surrounding the I*RX* genes on 5p in the expression of FIS (Miller *et al.* 2006). The *IRX* genes are surrounded by a large amount of CNEs, which share sequence similarity between regions on the same chromosome, as well as with regions on 16q (Bejerano *et al.* 2004; de la Calle-Mustienes *et al.* 2005; McEwen *et al.* 2006; Sandelin *et al.* 2004; Woolfe *et al.* 2005). Several vertebrate CNE regions have been tested *in vivo* in zebrafish and mouse, and many of these have been shown to function as tissue-specific enhancers (de la Calle-Mustienes *et al.* 2005; Goode *et al.* 2005; McEwen *et al.* 2006; Nobrega *et al.* 2003; Woolfe *et al.* 2005).

In this study, we examined whether the *IRX* genes or the surrounding regulatory elements are involved in the development of FIS by sequencing the exons and the CNEs located 500 kb upstream and downstream from *IRX1*, *IRX2*, and *IRX4*. Intrafamilial tests of association were used to identify significantly associated SNVs. In addition, a SNV in a highly conserved noncoding region associated with FIS in one family was selected to test for functional significance. Zebrafish transgenesis was carried out in order to determine if the CNE surrounding this SNV, rs117273909, acts as an enhancer *in vivo*, and if the alternate allele affects the enhancer function.

## Materials and Methods

### Subjects

All probands and their relatives were clinically characterized by a single orthopedic surgeon. Written informed consent was obtained for all study participants, in accordance with the Institutional Review Board of the participating institutions.

### Primer design for genes and conserved noncoding elements

Primers covering the *IRX* exons genes, the CNEs, and a long intergenic nonprotein RNA in the linkage region, *LOC285577*, were designed using Primer3 software (http://frodo.wi.mit.edu/cgi-bin/primer3/primer3_www.cgi). CNEs were selected for sequencing if they had a LOD score > 100 based on the PhastCons Placental Mammal Conserved Elements, 28-way Multiz Alignment (http://www.genome.ucsc.edu), and 500 kb ± from *IRX1*, *IRX2*, and *IRX4* (Supplemental Material, Figure S1 and Table S1). For rs117273909, genotyping of the controls was performed by sequencing. Sequences of primers are available on request.

### PCR and sequencing

For mutational analysis of the genes, PCR was performed on DNA from 53 individuals from seven FIS families from the KS clinical subset using the HotStarTaq amplification protocol (Qiagen). For the sequencing of CNEs and *LOC285577*, PCR from 46 of these individuals was carried out using the KAPA 2G Fast HS ReadyMix PCR Kit (KAPA Biosystems, Wilmington, MA). The reactions were analyzed on 3730 DNA Sequencers (Applied Biosystems, Grand Island, NY).

DNA isolated from blood samples of 100 controls consisting of individuals who married into FIS families, and who did not have FIS, were amplified with rs117273909 primers using GeneAmp High Fidelity PCR System (Applied Biosystems, Grand Island, NY). The products were sequenced on an Applied Biosystems / Hitachi 3730 Genetic Analyzer. Sequencing analysis was performed using Sequencing Analysis version 5.2, and Sequence Scanner version 1.0 (both from Applied Biosystems, Grand Island, NY). Alignments of DNA sequences were done with SeqScape (Applied Biosystems, Grand Island, NY), Sequencher (Gene Codes Corporation, Ann Arbor, MI), and CodonCode Alignment software (v 3.7.1.1).

### Statistical methods

Data cleaning was carried out on 344 CNE SNVs and 70 insertions/deletions from 46 individuals. Individuals with a genotype missing rate > 10%, and variants (SNVs, insertions and deletions) with either a Polyphred (v6.11) value < 99, a missing rate > 10%, and/or two or more Mendelian inconsistencies were removed. The Polyphed program identifies heterozygous single nucleotide substitutions, and assigns scores ranging from 99 to 0 to each heterozygous site, where a score of 99 indicates a very good fit and stands for a true positive rate of > 97%. Mendelian inconsistencies were tested using PEDCHECK (O’Connell and Weeks 1998). SNVs that became monomorphic after these steps were removed. Data cleaning reduced the number of individuals for analysis to 38, the number of SNVs to 197, and the number of insertions/deletions to 40. Of the remaining individuals, 22 had scoliosis (of which 14 had KS), 12 were unaffected and four had no curvature information. Tests of Hardy-Weinberg equilibrium (Wigginton *et al.* 2005) identified one SNV (hg19, chr5:3188028) with a *P*-value < 0.0001, which was retained for analysis because our sample was ascertained for familial idiopathic scoliosis and removal of SNVs in this situation can remove causative SNVs.

All SNVs were tested for association to the quantitative trait using ASSOC [S.A.G.E., v.6.0.1], a likelihood-based test of association that compares the likelihood of the data in models with and without a marker, and uses the phenotype and genotype information of the entire family. The degree of lateral curvature was analyzed as a quantitative phenotype, and genotypic (*a/a*, *a/A*, or *A/A*) and allelic (presence of minor allele) tests of association were performed.

### Cloning of putative regulatory elements into zebrafish enhancer detection vector

Site-directed mutagenesis was used to change the wild type rs117273909 C allele to the variant T allele (Bioinnovatise, Rockville, MD). Four constructs (198bp C allele, 198bp T allele, 687bp C allele, and 687bp T allele) were cloned into the zebrafish enhancer detection (ZED) vector (Bessa *et al.* 2009), which has insulators that prevent false positive expression due to position effects. Tol2 mRNA was synthesized with the mMessage mMachine SP6 kit (Ambion, Grand Island, NY) using *Not*I linearized pCS2FA-transposase vector as template DNA (Kwan *et al.* 2007).

### Zebrafish transgenesis

Microinjections of all four constructs were performed into one- to two-cell stage zebrafish embryos using 25–45 pg of plasmid DNA mixed with 50 pg of Tol2 mRNA. Embryos were incubated at 28° with 0.003% PTU (1-phenyl 2-thiourea) to suppress pigmentation. Embryos with transgene integration were identified by red fluorescent protein (RFP) expression in the skeletal muscle at 48 hr postfertilization (hpf), and green fluorescent protein (GFP) expression was observed in RFP positive embryos from 48 hpf to 5 d post fertilization (dpf). Embryos positive for both RFP and GFP were grown to adulthood. Several germline transmitting founders were identified for each construct, and their progeny were evaluated for patterns of GFP expression from 48 hpf to 5 dpf. Embryos from F2 generation of germline transmitting founders were also evaluated for GFP expression by crossing F1 adult fish with wildtype fish. The only difference in sequence between the C and T allele constructs was at the rs117273909 locus. Screening and imaging of embryos were performed using the Zeiss SteREO Lumar.V12 stereomicroscope with an AxioCam HRC color camera or Zeiss Axio Observer Z1 inverted microscope with an AxioCam MRm black and white camera.

### Data availability

The authors state that all data necessary for confirming the conclusions presented in the article are represented fully within the article.

## Results

### Variant analysis of IRX genes exons and CNEs

Sequencing of the *IRX1*, *IRX2*, and *IRX4* exons in 53 individuals did not identify any functional sequence variants. Sequencing of the CNEs surrounding the IRX genes in 46 individuals identified 197 SNVs suitable for analysis, of which 16 were ± 500 kb of *IRX4*, 70 were ± 500 kb of *IRX2*, and 160 were ± 500 kb of *IRX1*, with eight SNVs overlapping the conserved regions of *IRX4* and *IRX2* and 41 SNVs overlapping the conserved regions of *IRX1* and *IRX2*. Of the 197 SNVs, 23 were novel.

**Figure 1:**
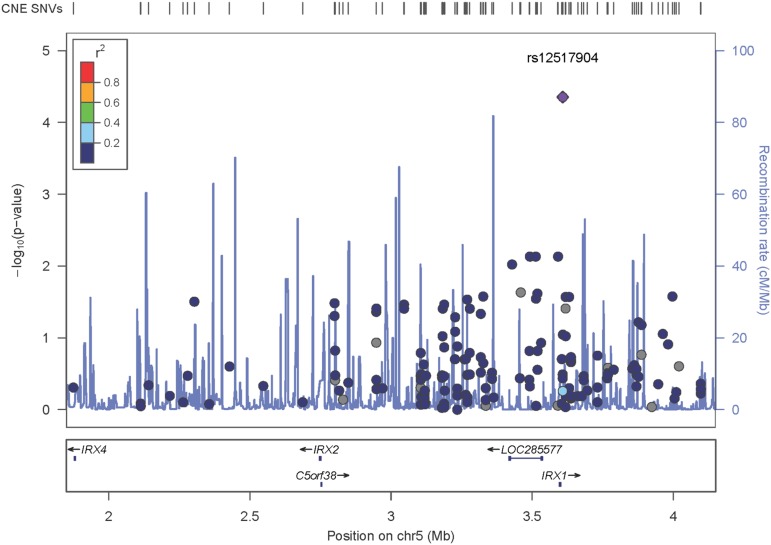
Regional allelic association plots of SNVs in CNEs surrounding *IRX* genes. The position of each SNV is indicated by the circles; diamond indicates the SNV with the most significant association (rs12517904, *P*-value = 0.00004). The color of the circles is the linkage disequilibrium (*r*^2^) between each SNV and rs12517904, indicated by the color scale on the left. Gray circles indicate a lack of linkage disequilibrium between the SNV and rs12517904. Genes in the region are indicated in the lower box. Recombination rates (cm/Mb) are depicted by the purple vertical lines. The plot was created using Locus Zoom (https://statgen.sph.umich.edu/locuszoom).

Intrafamilial tests of association between the genotypes of the 197 SNVs and the quantitative trait (scoliotic curvature) resulted in 30 SNVs (Table 1) with nominal significance (*P*-values < 0.05). Only rs12517904 (*P*-value = 0.00004, Figure 1) was significant after adjusting for multiple testing (Bonferroni correction *P*-value = 0.00013). No association was identified with the 40 insertions/deletions.

**Table 1:** Association analysis results (*P*-values < 0.05)

SNP	Position*^a^*	MAF*^b^*	Allelic Association	Genotypic Association
rs67250895	2303160	0.22	0.03124	0.14792
rs139215365	2427684	0.07	0.25035	0.03999
rs16870466	2799648	0.11	0.04946	0.05745
rs1497457	2799759	0.31	0.03285	0.19374
rs2934527	2947498	0.48	0.04338	0.09179
rs2934528	2947877	0.32	0.03903	0.07172
rs183225473	3045672	0.04	0.03913	0.03913
rs146531224	3046004	0.20	0.03438	0.03438
rs117273909	3182971	0.44	0.03913	0.03913
rs111916055	3187995	0.03	0.03438	0.03438
rs16871553	3269602	0.39	0.02924	0.02924
rs73733752	3278780	0.54	0.03913	0.03913
rs61712864	3317668	0.18	0.04626	0.25960
rs73733769	3326276	0.03	0.02653	0.02653
rs73032754	3428448	0.04	0.00948	0.00965
novel	3459187	0.13	0.03913	0.03913
rs117494736	3459291	0.10	0.02326	0.02326
rs537539844	3491460	0.03	0.03893	0.03893
rs35450818	3491486	0.03	0.00737	0.03819
rs34560950	3512343	0.50	0.00737	0.00737
rs62336074	3513171	0.47	0.02838	0.02838
rs35155570	3518273	0.03	0.02409	0.02409
rs828332	3591325	0.03	0.00737	0.00737
rs12517904	3608064	0.03	0.00004	0.16992
rs76205392	3617948	0.03	0.03893	0.03893
rs71577554	3618245	0.03	0.02686	0.02686
rs10475220	3618387	0.22	0.02686	0.02686
rs10475221	3618393	0.03	0.02686	0.02686
rs78040936	3630480	0.20	0.02686	0.02686
novel	4003967	0.04	0.02645	0.02645

a Map positions obtained from NCBI (GRCh37/hg19).

b Maximum likelihood estimate of minor allele frequency from founders (FREQ [S.A.G.E., v6.0.1]).

We searched for variants cosegregating with KS. In one family, the genotypes of nine SNVs differed from the reference allele in all individuals affected with KS, and two out of three individuals with FIS (Table 2); unaffected family members, and all other individuals genotyped for this study, did not differ from the reference allele for any of these nine SNVs. The one family member with FIS that did not differ from the reference allele had a scoliotic curve of 36°, and no hyperkyphosis. Three of these SNVs were located in highly conserved regions, based on whole-genome alignment of vertebrates. Of these three, only rs117273909 (*P*-value = 0.039 for association analysis using all families genotyped in this study) was found to be conserved in all 96 vertebrate species in which this SNV and the corresponding CNE were present (www.genome.ucsc.edu). Rs117273909 is located in the ∼841 kb gene desert between *IRX1* (413 kb downstream) and *IRX2* (431 kb downstream), and thus could function as an enhancer for either *IRX1* or *IRX2*, or for both.

**Table 2:** SNV genotypes for family 1

SNV	bp	Conserved Region*^a^*	*P*-Value*^b^*	Alleles*^c^*	MAF*^d^*	Unaffected			Scoliosis			Kyphoscoliosis			
rs114080324	2947499	Yes	0.116	G/A	0.006	GG	GG	GG	—	GG	AG	AG	AG	AG	AG
rs183225473	3045672	No	0.039	G/C	0.006	GG	GG	GG	CG	GG	CG	CG	CG	CG	CG
rs10068728	3045983	Yes	0.034	T/G	0.27	TT	TT	TT	TG	TT	—	TG	TG	TG	TG
rs146531224	3046004	No	0.039	G/A	0.006	GG	GG	GG	AG	GG	AG	AG	AG	AG	AG
rs117273909	3182971	Yes	0.039	C/T	0.005	CC	CC	CC	CT	CC	CT	CT	CT	CT	CT
rs111916055	3187995	No	0.034	A/G	0.028	AA	AA	AA	AG	AA	—	AG	AG	AG	AG
Novel	3188045	No	0.083	T/G	NA	TT	TT	TT	AT	TT	—	AT	AT	—	AT
rs16871553	3269602	No	0.029	C/T	0.109	TT	TT	TT	CT	TT	CT	CT	CT	CT	—
rs73733752	3278780	No	0.039	A/G	0.03	AA	AA	AA	AG	AA	AG	AG	AG	AG	AG

a Based on GERP score > 2 (www.genome.uscs.edu).

b *P*-values the same for genotypic and allelic association tests, using all families in study.

c Reference allele/alternate allele.

d Minor allele frequency, from dbSNP website (www.ncbi.nlm.nih.gov/SNP).

We determined the frequency of the variant T allele of rs117273909 in a Caucasian population by sequencing 100 controls consisting of individuals who married into our FIS families, and who did not have a history of FIS. Of these 100 controls, 90 matched the reference sequence (C), one was heterozygous (C/T), and nine failed to amplify, resulting in a T allele frequency of 0.00515. In the 1000 Genomes Project (www.1000genomes.org), rs117273909 was found to be C/T in 27 of 5008 genotypes (T allele frequency of 0.00539), and heterozygous A/T in three out of 5008 genotypes.

Based on the association with KS (*P*-value = 0.039), the presence of a nonreference allele cosegregation with KS in a single family, the fact that rs117273909 is located in a noncoding fragment (element_603, hg19 chr5:3182218-3183271,VISTA Enhancer Browser, http://enhancer.lbl.gov), which drives expression in the hindbrain in five out of 10 transgenic mice embryos (Visel *et al.* 2007), and the highly conserved nature of this SNV, we gave this SNV priority in our effort to determine if this SNV was functional. We then used zebrafish transgenesis to evaluate the effect of a C to a T allele substitution (rs117273909).

### Regulatory activity of CNE containing rs117273909

We performed zebrafish transgenesis to determine if changing the allele at rs117273909 from a C (wild type allele) to a T would result in a change in its regulatory activity. Tena *et al.* (2011) tested a 1172bp fragment encompassing rs117273909, which did not show any regulatory activity in *Xenopus*, possibly due to the presence of repressors: therefore, we chose to test smaller fragments for regulatory activity. For the zebrafish transgenesis assay, the wild type (C allele) and variant (T allele) versions of a 198 bp fragment (hg19, chr5:3182938-3183135), and an overlapping larger 687 bp fragment (hg19, chr5:3182466-3183152), both selected based on strong conservation across vertebrates, were tested for regulatory activity (Figure S2). Despite strong RFP expression, no consistent GFP expression patterns were observed in the embryos injected with any of the four constructs (Figure S3).

**Figure 2:**
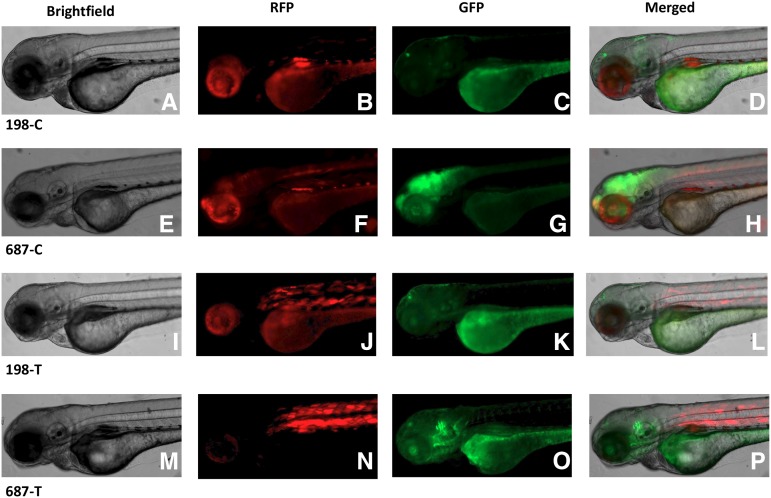
Patterns of GFP expression in F1 embryos at 72 hpf. Images of lateral views of representative embryos for each of the four constructs are shown in Brightfield (A, E, I, and M), RFP expression (B, F, J, and N), GFP expression (C, G, K, and O) and merged image of all three images (D, H, L, and P). In all images, embryos are oriented with their anterior to the left.

Due to the mosaic nature of the transgene expression during transient transgenesis, we generated multiple stable transgenic lines for all four constructs by screening founders for germline transmission. Variable GFP expression patterns, ranging from weak and diffused expression to strong and specific expression, were observed in the F1 and F2 progeny of multiple germline transmitting founders for each of the four constructs (Figure 2, A–P). These data may indicate positional effects of the transgene integration in independent founders for each of the four constructs. The specific GFP expression patterns were observed in pineal gland, pharyngeal arches, and brain. However, there was no association of these expression patterns with any particular construct.

## Discussion

Our results suggest that noncoding fragments as small as 198 bp can act as regulatory elements in zebrafish, which is in contrast to a report that a 1172 bp fragment (TA3235, 131:818307–820479 bp, xenTrot2) overlapping our 198 bp and 697 bp fragments did not drive expression in *Xenopus* (Tena *et al.* 2011). At present, there are no guidelines regarding the identification of regulatory regions, the size of these regions, or the type of activity present (repressors *vs.* enhancers). We were not able to determine in this study whether this regulatory region plays a role in the expression of FIS. Although we observed a strong expression trend with one of the two alleles at rs117273909, we did not observe a common pattern of expression among multiple lines, and thus could not rule out position effects. GFP expression occurring in different regions (the pharyngeal arches, pineal gland and brain) could be due to positional effects, something the ZED vector should minimize, or could be the result of the involvement of this regulatory region with a homeobox gene. The expression of our conserved fragment in the pineal gland is of interest, since pinealectomised chickens develop scoliosis (Machida *et al.* 1993).

There appeared to be differences in the level of expression and the timing of GFP expression between the wild type and alternate allele at rs117273909. de la Calle-Mustienes *et al.* (2005) noticed that DNA sequence differences in zebrafish and mouse CNEs did not result in differences in where expression occurred, but affected the level and/or timing of the transcription. The change in timing and regulation may be due to the sequence degeneration, which interferes with loop formation. Tena *et al.* (2011) identified a three-dimensional architecture that forms through CCCTC-binding, present in the *Irx* clusters of mice, zebrafish, and *Xenopus*, which brings the *Irx1/3* and *Irx2/5* promoters together, and demonstrated that *cis*-regulatory elements in the *Irx* clusters interact with more than one *Irx* promoter, up to distances of 1.6 Mb. This looping mechanism may help facilitate the delivery of RNA polymerase, transactivators, and transcription factors to the promoter to the right tissue at the correct time.

In summary, tests of association identified several significant SNVs that may lie in regulatory regions that influence gene expression. These SNVs may play a role in the phenotype, but the relevance of each individual SNV can be determined only by *in vitro* and *in vivo* assays. It is also possible that unidentified SNVs in highly conserved regulatory regions further upstream from the *IRX* family may disrupt developmental patterning and be responsible for the variation of the degree of lateral and thoracic curvature in individuals in these families. Future work will focus on completely sequencing the region with the most significant association, as well as performing additional *in vivo* assays of regulatory regions surrounding the other associated SNVs identified in this study, including rs1251709, and the SNVs in *LOC285577*, of which little is known to date. Once we have a better understanding of what tissues are involved in the scoliosis phenotype, we will be able to define the regulatory regions using both *in vitro* and *in vivo* assays.

## 

## Supplementary Material

Supplemental Material
