# Supplementary material for: Evaluation of IRX Genes and Conserved Noncoding Elements in a Region on 5p13.3 Linked to Families with Familial Idiopathic Scoliosis and Kyphosis
Source: G3 (Bethesda). 2016 Apr 12;6(6):1707–12. doi: 10.1534/g3.116.029975 (PMC4889666; doi:10.1534/g3.116.029975)
Supplement: Supplemental Material [file supp_6_6_1707__index.html]

Evaluation of IRX Genes and Conserved Noncoding Elements in a Region on 5p13.3 Linked to Families with Familial Idiopathic Scoliosis and Kyphosis — Supplemental Material 

# Evaluation of *IRX* Genes and Conserved Noncoding Elements in a Region on 5p13.3 Linked to Families with Familial Idiopathic Scoliosis and Kyphosis

## Supplemental Material for Justice *et al.*, 2016

**Files in this Data Supplement:**

- Figure S1 - Regional plots of sequenced CNEs and the amplimers used to capture them surrounding *IRX4* (A), *IRX2* (B) and *IRX1* (C). (.pdf, 144 KB)
- Figure S2 - Schematic representation of the region surrounding the conserved fragments used for zebrafish transgenesis. (.pdf, 268 KB)
- Figure S3 - Somatic GFP expression in embryos injected with (A, B) 198bp C allele fragment, (C, D) 198bp T allele fragment, (E, F) 687 bp C allele fragment, (G, H) 687bp T allele fragment. (.pdf, 206 KB)
- Table S1 - Position (GRCh37/hg19) of amplimers and the CNEs they target. (.pdf, 263 KB)
