# Supplementary material for: Evaluation of IRX Genes and Conserved Noncoding Elements in a Region on 5p13.3 Linked to Families with Familial Idiopathic Scoliosis and Kyphosis
Source: G3 (Bethesda). 2016 Apr 12;6(6):1707–12. doi: 10.1534/g3.116.029975 (PMC4889666; doi:10.1534/g3.116.029975)
Supplement: Supplemental Material [file supp_g3.116.029975_FigureS1.pdf]

Figure S1.

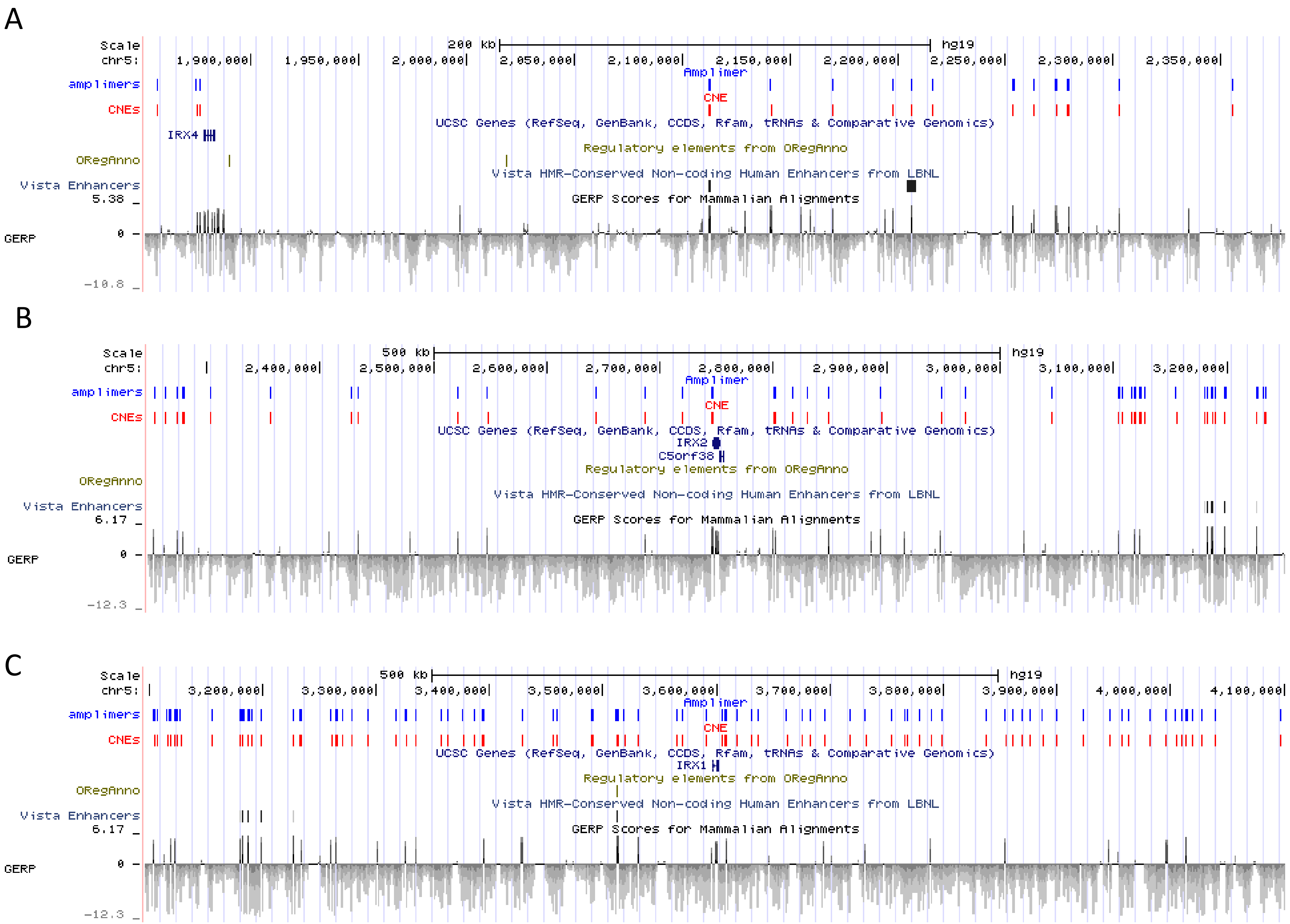
A
Scale
chr5:
200 kb
hg19
1,900,000
1,950,000
2,000,000
2,050,000
2,100,000
2,150,000
2,200,000
2,250,000
2,300,000
2,350,000
amplimers
Amplimer
CNEs
CNE
UCSC Genes (RefSeq, GenBank, CCDS, Rfam, tRNAs & Comparative Genomics)
IRX4
Regulatory elements from ORegAnno
ORegAnno
Vista HMR-Conserved Non-coding Human Enhancers from LBNL
Vista Enhancers
5.38
GERP Scores for Mammalian Alignments
GERP
0
-10.8
B
Scale
chr5:
500 kb
hg19
2,400,000
2,500,000
2,600,000
2,700,000
2,800,000
2,900,000
3,000,000
3,100,000
3,200,000
amplimers
Amplimer
CNEs
CNE
UCSC Genes (RefSeq, GenBank, CCDS, Rfam, tRNAs & Comparative Genomics)
IRX2
C5orf38
Regulatory elements from ORegAnno
ORegAnno
Vista HMR-Conserved Non-coding Human Enhancers from LBNL
Vista Enhancers
6.17
GERP Scores for Mammalian Alignments
GERP
0
-12.3
C
Scale
chr5:
500 kb
hg19
3,200,000
3,300,000
3,400,000
3,500,000
3,600,000
3,700,000
3,800,000
3,900,000
4,000,000
4,100,000
amplimers
Amplimer
CNEs
CNE
UCSC Genes (RefSeq, GenBank, CCDS, Rfam, tRNAs & Comparative Genomics)
IRX1
Regulatory elements from ORegAnno
ORegAnno
Vista HMR-Conserved Non-coding Human Enhancers from LBNL
Vista Enhancers
6.17
GERP Scores for Mammalian Alignments
GERP
0
-12.3

Figure S1 Regional plots of sequenced CNEs and the amplimers used to capture them surrounding *IRX4* (A), *IRX2* (B) and *IRX1* (C). Map positions are from GRCh37/hg19. Regulatory regions annotated with ORegAnno or Vista are depicted (genome.ucsc.edu). Evolutionary constraint is indicated using Genomic Evolutionary Rate Profiling (GERP) scores.
