# Supplementary material for: Evaluation of IRX Genes and Conserved Noncoding Elements in a Region on 5p13.3 Linked to Families with Familial Idiopathic Scoliosis and Kyphosis
Source: G3 (Bethesda). 2016 Apr 12;6(6):1707–12. doi: 10.1534/g3.116.029975 (PMC4889666; doi:10.1534/g3.116.029975)
Supplement: Supplemental Material [file supp_g3.116.029975_FigureS2.pdf]

Figure S2.

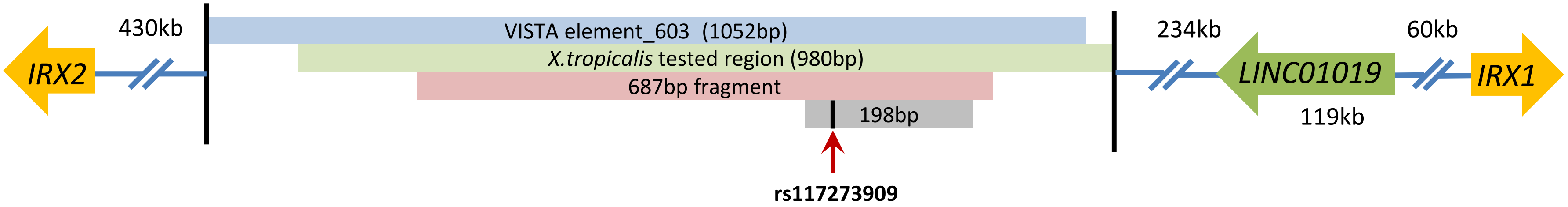

Figure S2 Schematic representation of the region surrounding the conserved fragments used for zebrafish transgenesis. The gray box corresponds to the 198bp fragment, and the red box representing the 687bp fragment. The location of rs117273909 is indicated with a red arrow. The noncoding fragment found to drive expression in mice (VISTA element_603, http://enhancer.lbl.gov) and the sequence corresponding to the fragment tested in X.tropicalis, which did not drive expression (Tena et al. 2011), are included here for comparison to the 198bp and 687bp fragments.
