# Supplementary material for: Evaluation of IRX Genes and Conserved Noncoding Elements in a Region on 5p13.3 Linked to Families with Familial Idiopathic Scoliosis and Kyphosis
Source: G3 (Bethesda). 2016 Apr 12;6(6):1707–12. doi: 10.1534/g3.116.029975 (PMC4889666; doi:10.1534/g3.116.029975)
Supplement: Supplemental Material [file supp_g3.116.029975_FigureS3.pdf]

Figure S3.

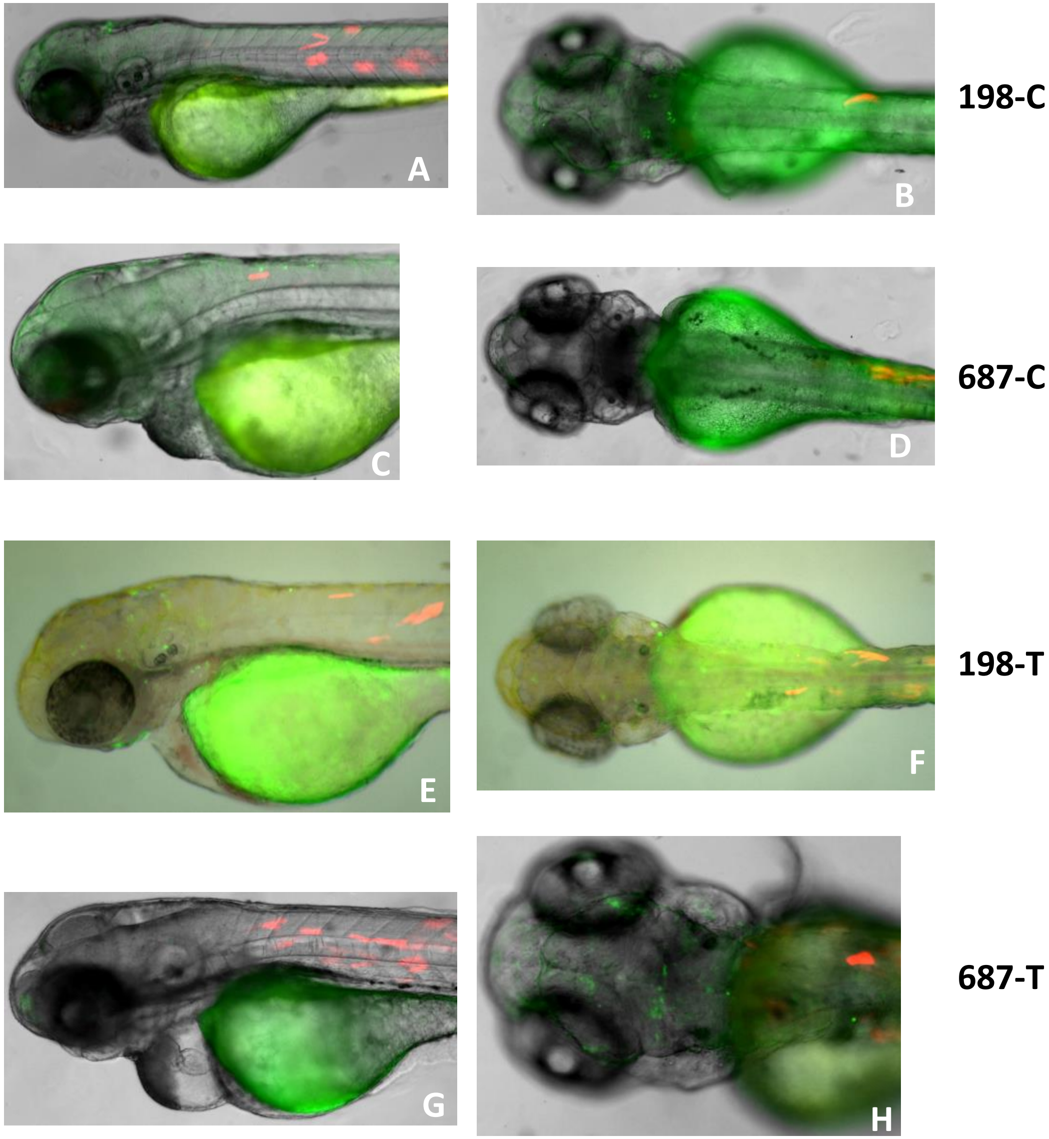

Figure S3 Somatic GFP expression in embryos injected with (A, B) 198bp C allele fragment, (C, D) 198bp T allele fragment, (E, F) 687 bp C allele fragment, (G, H) 687bp T allele fragment. Merged images (Brightfield, GFP and RFP) of two representative embryos are shown for each construct. Embryos are oriented with their anterior to the left. (A, C, E, G) lateral views and (B, D, F, H) dorsal views.
