# Supplementary material for: Evaluation of IRX Genes and Conserved Noncoding Elements in a Region on 5p13.3 Linked to Families with Familial Idiopathic Scoliosis and Kyphosis
Source: G3 (Bethesda). 2016 Apr 12;6(6):1707–12. doi: 10.1534/g3.116.029975 (PMC4889666; doi:10.1534/g3.116.029975)
Supplement: Supplemental Material [file supp_g3.116.029975_TableS1.pdf]

Table S1. Position (GRCh37/hg19) of amplimers and the CNEs they target.

Amplimer Regions			CNEs	
bp start	bp end	Passed QC	bp start	bp end
1855935	1856624	Y	1856012	1856162
1874215	1874844	Y	1874705	1875194
1874908	1875625	N		
1874929	1875577	N		
1875884	1876614	Y	1875963	1876420
1884332	1885066	N	1884358	1884895
1884358	1885019	N		
1886739	1887443	N	1886905	1887421
1886880	1887583	N		
1887255	1887928	N		
2112338	2112861	Y	2112466	2113237
2112693	2113379	Y		
2140820	2141511	Y	2140936	2141306
2169746	2170420	Y	2169810	2169944
2197414	2198078	Y	2197516	2197794
2206198	2206953	Y	2206223	2206617
2215637	2216341	N	2215811	2215987
2215764	2216485	Y		
2253158	2253627	Y	2253248	2253694
2253158	2253627	Y		
2253444	2254155	N		
2253444	2254155	Y		
2263178	2263879	Y	2263308	2263475
2273200	2273939	Y	2273374	2273665
2273200	2273939	Y		
2278397	2279092	Y	2278519	2278644
2279112	2279756	Y	2279172	2279306
2279112	2279756	Y		
2302573	2303283	Y	2302693	2302937
2302573	2303283	Y		
2355333	2356022	Y	2355411	2355522
2427214	2427919	Y	2427354	2427541
2432849	2433503	N	2433018	2433368
2432921	2433669	Y		
2521064	2521817	Y	2521088	2521367
2547265	2548001	Y	2547376	2547518
2643153	2643870	Y	2643291	2643591
2686551	2687234	Y	2686633	2686838
2718526	2719208	Y	2718690	2718881
2744973	2745710	Y	2745040	2745475
2746141	2746846	Y	2746281	2746660
2751268	2751876	N	2751271	2751902
2751773	2752304	N		
2799461	2800166	Y	2799510	2800006
2801399	2802094	Y	2801556	2801870
2816070	2816659	Y	2816243	2816515
2829363	2830067	Y	2829419	2829634
2848447	2849132	Y	2848528	2848888
2894377	2895084	Y	2894455	2894774
2947409	2948096	Y	2947463	2947886
2969243	2969968	Y	2969354	2969579
3045582	3046281	Y	3045674	3045942

3103820	3104582	Y	3103963	3104671
3104476	3105156	Y		
3106817	3107506	Y	3106873	3106969
3115517	3115944	Y	3115745	3115846
3117877	3118564	Y	3118036	3118164
3118493	3119179	Y	3118618	3119264
3119075	3119775	Y		
3122357	3123038	Y	3122429	3122970
3123408	3124153	Y	3123946	3124403
3124185	3124925	Y		
3127289	3127993	Y	3127466	3127574
3154708	3155397	Y	3154824	3154927
3179679	3180382	Y	3179855	3180348
3180193	3180889	Y		
3182176	3182863	Y	3182341	3183128
3182734	3183336	Y		
3186431	3187129	Y	3186570	3187850
3186999	3187685	Y		
3187567	3188196	Y		
3189564	3190310	Y	3189696	3189751
3197946	3198718	Y	3198055	3198683
3198619	3199289	Y		
3226128	3226776	Y	3226301	3227205
3226622	3227274	Y		
3231893	3232662	Y	3232229	3232307
3233942	3234654	Y	3234009	3234156
3259970	3260676	Y	3260136	3260616
3264152	3264915	Y	3264289	3264485
3264942	3265673	Y	3264927	3264997
3269558	3270160	Y	3269635	3269708
3278276	3278806	Y	3278361	3278520
3292225	3292942	Y	3292436	3292553
3317398	3318100	Y	3317456	3317542
3325623	3326308	Y	3325761	3326322
3326083	3326730	Y		
3334660	3335389	Y	3334777	3335050
3357261	3357959	Y	3357396	3357767
3363061	3363753	Y	3363166	3363410
3376086	3376679	Y	3376261	3376422
3386147	3386808	Y	3386317	3386464
3393690	3394381	Y	3393797	3394204
3394830	3395540	Y	3394906	3395416
3427994	3428673	Y	3428124	3428559
3455416	3456136	Y	3455600	3455834
3458814	3459521	Y	3458989	3459106
3489530	3490209	Y	3489666	3489937
3491144	3491869	Y	3491204	3491247
3511131	3511892	Y	3511507	3513870
3512212	3512982	Y		
3512791	3513461	Y		
3513323	3514014	Y		
3518238	3518904	Y	3518332	3518417
3530705	3531426	Y	3530767	3531379
3564307	3565053	Y	3564456	3564877
3569508	3570199	Y	3569600	3569741

3589958	3590690	N	3590136	3590241
3589963	3590615	Y		
3590716	3591422	Y	3590820	3591127
3595589	3596197	N	3595775	3596096
3595660	3596256	N		
3604559	3605228	Y	3604701	3604849
3604640	3605343	N		
3607241	3607932	Y	3607388	3607464
3607910	3608604	Y	3608004	3609004
3608481	3609139	Y		
3617890	3618590	Y	3618033	3618232
3630120	3630827	N	3630271	3630431
3630209	3630894	Y		
3636342	3637042	N	3636457	3636676
3636390	3637106	Y		
3661475	3662173	Y	3661617	3661813
3674780	3675476	Y	3674899	3674954
3681980	3682496	Y	3682153	3682317
3694418	3695175	Y	3694513	3694761
3717349	3718105	Y	3717503	3717614
3730618	3731247	Y	3730786	3730862
3753198	3753917	Y	3753328	3753433
3765202	3765865	Y	3765352	3765429
3765275	3765961	N		
3767853	3768625	Y	3768295	3768453
3778169	3778866	Y	3778277	3778400
3788288	3788986	Y	3788352	3788406
3797746	3798425	Y	3797883	3797948
3836862	3837596	Y	3836923	3837144
3853995	3854691	Y	3854074	3854533
3860806	3861506	Y	3860986	3861036
3868532	3869140	Y	3868666	3868843
3876205	3876881	Y	3876331	3876419
3886774	3887184	Y	3886903	3887040
3899142	3899831	Y	3899328	3899400
3922562	3923330	Y	3922677	3922773
3945821	3946419	Y	3945950	3946059
3956713	3957434	Y	3956775	3956907
3962253	3963025	Y	3962734	3962829
3981065	3981699	Y	3981173	3981363
3995880	3996543	Y	3996053	3996492
4003867	4004463	Y	4004313	4004481
4009933	4010634	Y	4010062	4010185
4013606	4014077	Y	4013683	4014372
4013855	4014565	Y		
4019099	4019768	Y	4019177	4019473
4027190	4027899	Y	4027351	4027590
4039051	4039756	Y	4039210	4039489
4096559	4097043	Y	4096713	4096843
